# Implementation of Multimodality Therapy and Minimally Invasive Surgery: Short- and Long-term Outcomes of Gastric Cancer Surgery in Medium-Volume Center

**DOI:** 10.1007/s11605-022-05437-3

**Published:** 2022-08-24

**Authors:** Anna Junttila, Olli Helminen, Valtteri Kairaluoma, Anne Mattila, Eero Sihvo, Johanna Mrena

**Affiliations:** 1 Department of Surgery, Central Finland Central Hospital, Keskussairaalantie 19 40620, Jyväskylä, Finland; 2 Division of Digestive Surgery and Urology, Turku University Hospital, Turku, Finland; 3 Surgery Research Unit, Medical Research Center Oulu, Oulu University Hospital and University of Oulu, Oulu, Finland

**Keywords:** Gastric cancer, Guideline, Perioperative treatment, Lymph node yield, Minimally invasive surgery, Laparoscopic gastrectomy

## Abstract

**Background:**

Multimodal treatment of gastric cancer includes careful preoperative staging, perioperative oncological treatment, and selective minimally invasive approach. The aim was to evaluate whether this approach improves short- and long-term outcomes in operable gastric cancer.

**Methods:**

This study included 181 gastric cancer patients who underwent curative intent surgery in Central Finland Central Hospital between years 2005 and 2021 for gastric or esophagogastric junction adenocarcinoma. Those 65 patients in group 1 operated between years 2005–2010 had open surgery with possible adjuvant therapy. During the second period including 58 patients (2011–2015), perioperative chemotherapy and minimally invasive surgery were implemented. The period, when these treatments were standard practise, was years 2016–2021 including 58 patients (group 3). Outcomes were lymph node yield, major complications and 1- and 3-year survival rates.

**Results:**

Median lymph node yield increased from 17 in group 1 and 20 in group 2 to 23 in group 3 (*p* < 0.001). Major complication rates in groups 1–3 were 12.3%, 32.8%, and 15.5% (group 1 vs. group 2, *p* = 0.007; group 2 vs. group 3, *p* = 0.018), respectively. Overall 1-year survival rates between study groups 1–3 were 78.5% vs. 69.0% vs. 90.2% (*p* = 0.018) and 3-year rates 44.6% vs. 44.8% vs. 68.1% (*p* = 0.016), respectively. For overall 3-year mortality, adjusted hazard ratio (HR) was 1.02 (95%CI 0.63–1.66) in group 2 and HR 0.37 (95%CI 0.20–0.68) in group 3 compared to group 1.

**Conclusions:**

In medium-volume center, modern multimodal therapy in operable gastric cancer combined with minimally invasive surgery increased lymph node yield and improved long-term survival without increasing postoperative morbidity.

## Introduction

Worldwide of all cancers, fifth in incidence (5.6%), and fourth (7.7%) in cancer-related mortality is gastric cancer (GC).^1^ For GC, the basis of curative treatment is surgery.^2^ Previously, surgery was the primary treatment option followed, if appropriate, by postoperative chemo- or radiotherapy.^3^

Today, upfront surgery is performed only for patients with early stage disease.^2^ For ≥ stage IB disease, multimodal approach with perioperative treatment is considered as a standard treatment,^2^ improving survival and R0 resection rate.^2,4–6^ A complete neoadjuvant response is, however, achieved only in 6–20%.^7–9^ In real-world practise, many patients are elderly with comorbidities and, therefore, in increased risk of postoperative complications and not often eligible for the multimodal approach. Therefore, they are often excluded from randomized trials.^10^ Overall, up to 35% of GC patients are not able to complete perioperative treatment.^11^

According to ESMO guideline, laparoscopic approach for early GC is becoming the recommended option. The current recommendations in Western countries in medically fit patients favour D2 dissection and an excision of a minimum of 16 lymph nodes.^2,12^ Therefore, concerns of the ability of minimally invasive approach to yield insufficient number of lymph nodes in advanced GC exist.^2^ Asian studies focusing on laparoscopic partial gastrectomy for GC in high volume centers have shown better short-term outcomes and comparable long-term outcomes.^13^ In addition to a lower complication rate and to a comparable overall and disease-free survival, a similar lymph node yield was achieved in laparoscopic and open gastrectomy according to a recent meta-analysis ^13^. In this analysis, of 5061 patients, only 347 were, however, outside Asia.^13^ Two recent European RCTs comparing these approaches including mainly advanced stage patients requiring neoadjuvant chemotherapy revealed comparable lymph node yield, short-term, and 1-year outcomes.^14,15^

The aim of this study was to compare short- and long-term outcomes after the implementation of guideline-based modern perioperative treatment protocol and minimally invasive approach in a real-world setting.

## Materials and Methods

### Patients

Central Finland Central Hospital (CFCH) provides specialized care for the district of Central Finland with a population of 270, 000. This retrospective study included 181 patients who underwent curative intent surgery in CFCH between 2005 and 2021 for histologically confirmed gastric or esophagogastric (EG) junction adenocarcinoma. Only operations with curative intent were included in this study (during the study period eight patients underwent palliative resection and were excluded). Operative approach was either total, subtotal or distal gastrectomy, or esophagogastric resection. All clinical data including patient characteristics, preoperative staging, perioperative treatment, surgical details, lymph node yield, and short-term outcomes were obtained from medical records. In the risk evaluation, the Charlson Comorbidity Index ^16^ and ASA grade were used. Complication data was collected prospectively and re-reviewed retrospectively by the authors. Survival information and mortality data were confirmed from the nationwide and obligatory Cause of Death Registry from Statistics Finland. The follow-up ended on February 14, 2022. The median follow-up time was 24 (IQR 12–61) months. The study was approved by the hospital district.

### Study Groups

The patients were divided into three study groups according to operation years. Group 1 (*n* = 65) consisted of patients operated between years 2005–2010 in the era of open surgery and adjuvant therapy. In group 2, patients (*n* = 58) were operated between years 2011–2015 when perioperative chemotherapy protocol and minimally invasive surgery were adopted. Group 3 consisted of patients (*n* = 58) operated between years 2016–2021 after the perioperative chemotherapy and radical minimally invasive approach were considered as a standard of care in our unit.

### Outcomes

The primary outcomes were lymph node yield, major complication rate, and overall 1- and 3-year survival. The secondary outcomes were blood loss, R0 resection rate, reoperation rate, hospital stay, overall complication rate, and 30- and 90-day mortality rates.

### Preoperative Evaluation

The preoperative diagnostic and staging protocol consisted of endoscopy with biopsies, endoscopic ultrasound, body computed tomography (CT), and positron emission tomography-(PET-)CT. Endoscopic ultrasound was performed to selectively to assess the need for a perioperative treatment in a patient with an endoscopically small, superficial tumor, or the possibility of less invasive, endoscopic treatment. PET-CT was performed to all patients with an esophagogastric tumor and in case of a larger gastric tumor especially with those of an intestinal type cancer. Diagnostic laparoscopy was performed selectively to exclude peritoneal metastases or to clarify inconclusive radiological staging. Staging was classified according to the 8th edition of the UICC/AJCC TNM categories.^12^ This required recoding of all surgical patients accordingly.

Our center started minimally invasive esophageal surgery in 2012 with experienced surgeon implementing a proven protocol. This had also an impact on the gastric cancer patients’ preoperative evaluation protocol and a technically relatively easy transition from open to laparoscopic gastrectomy.^17^ Preoperative evaluation of exercise capacity was standardized and included a preclinical questionnaire, a history of physical performance and exercise testing by the stair-climbing test.^18^ The nutritional status was also evaluated.

### Perioperative Treatment

Before 2010 for gastric cancer, chemotherapy or chemoradiotherapy was used in eligible patients only as adjuvant treatment.^19^ From 2010 according to ESMO guidelines, patients with > T1b and/or > N0 tumors were evaluated for perioperative treatment.^2,20^ During the whole study period, > T2 and/or > N0 in Siewert type II esophagogastic tumors received either perioperative chemotherapy or preoperative chemoradiotherapy. After receiving perioperative treatment, patients were restaged before the surgery. The operation was performed usually after a 6-week recovery period.

### Operative Approach and Postoperative Care

#### Subtotal or Total Gastrectomy: Open and Laparoscopic Approaches

Gastrectomy was performed in a standardized manner ^2,19^ by specialized senior upper gastrointestinal surgeons. Subtotal gastrectomy was preferred in middle or distal-third tumors when macroscopic a minimum proximal margin of 5 cm (8 cm for diffuse adenocarcinoma) could be achieved. A standardized D2 lymphadenectomy, a technique well-established before this study,^21^ was performed according to Japanese Gastric Cancer Association (JGCA).^22^ In the era of open surgery, the indication of splenectomy was a tumor locating in the middle or upper part of a greater curvature. Resection of the pancreas or additional organs was performed only in selected cases when direct invasion was detected and R0 resection was considered achievable.

From 2016, the primary approach was 3D laparoscopy. In subtotal gastrectomy, all anastomoses were made by a laparoscopic linear stapler. After total gastrectomy esophagojejunostomy was made by a circular stapler via a laparoscopic gel port. Transoral OrVil™ and double-stapling anastomosis was mainly used. The principles of lymphadenectomy and radicality in the laparoscopic gastrectomy were similar as in open surgery, except in laparoscopic surgery omentectomy, splenectomy, and cholecystectomy were not routinely performed.

#### Esophagectomy

For EG junction tumors, a minimally invasive Ivor Lewis approach with intrathoracic anastomosis and en-bloc 2-field lymphadenectomy was our preferred procedure.^23^ Laparoscopy was performed in supine and thoracoscopy in left lateral position. Intrathoracic end-to-side anastomosis was performed using a circular stapler and was reinforced with an omental flap. A selective feeding jejunostomy tube placement and routine endoscopic pyloric dilatation were included in the operation.

#### Perioperative and Postoperative Care

Perioperative care included the assessment and optimization of medical risk factors, thromboprophylaxis with low-molecular-weight heparin and elastic stockings, prophylactic antibiotics, standard anesthesia with epidural analgesia, avoidance of hypothermia, and increased oxygen concentrations. A postoperative CT or fluorography with oral contrast was performed before oral fluid intake from two to five days after surgery.

For each patient who developed a postoperative complication within 30 days, a complication grade from 1 to 5 was assigned according to Clavien-Dindo Classification.^24^ The complications basic platform published by the Esophagectomy Complications Consensus Group (ECCG) and the Gastrectomy Complications Consensus Group (GCCG) was standardized according to suggestions and strictly used.^25,26^

After surgery and complementation of adjuvant therapy, patients were followed every 6 months for two years, and then once a year for up to 5 years at the surgical outpatient clinic.

### Statistical Analysis

Survival times were calculated from the date of surgery until the time of death or the end of follow-up (February 14, 2022). Kaplan–Meier survival curves were calculated according to the life table method to visualize the crude overall survival up to 3 years after surgery. Multivariable Cox regression was used for calculating hazard ratios (HRs) with 95% confidence intervals (CIs) of overall survival. Group 1 was used as the reference group in all analyses. The regression models were adjusted for potential confounding factors: sex (male / female), age (< 75 years, ≥ 75 years), Charlson Comorbidity Index (0–1, ≥ 2) and pathological stage (0–I, II, and III–IV). Total of six patients had missing Charlson Comorbidity Index. Multiple imputation was used to add these in regression model. Proportions, means, and median values of other measured variables were compared using the Chi-squared test and Mann–Whitney *U*-test as appropriate. All statistical analyses were conducted using IBM SPSS 26.0 (IBM corp., Armonk, NY, USA).

## Results

**Fig. 1:**
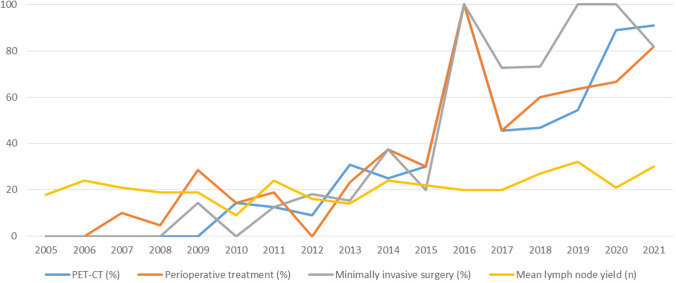
Changes over time in the rate of PET-CT (%), perioperative treatment (%), minimally invasive surgery (%), and mean lymph node yield (*n*)

Baseline information is shown in Table 1. No major differences in age, BMI, or Charlson Comorbidity Index were detected between the study groups. The rate of PET-CT staging increased from 1.5% in group 1 and 20.7% in group 2 to 63.8% in group 3 (*p* < 0.001). The perioperative treatment rate increased from 7.7% in group 1 and 20.7% in group 2 to 63.8% in group 3 (group 1 vs. group 3 and group 2 vs. group 3, *p* < 0.001. A total of 21 were planned to have preoperative and 33 perioperative treatment. Of those with intended preoperative treatment, 19 (90.5%) completed the planned regimen. Patients with perioperative treatment, 32 (97.0%) completed the preoperative phase and 24 (72.7%) completed also the postoperative phase. Between groups 1–3, the rate of minimally invasive approach was 1.5%, 17.2%, and 84.5% (*p* < 0.001), respectively. Changes over time in the rate of PET-CT, perioperative treatment, minimally invasive surgery, and lymph node yield are presented in Fig. 1.

**Table 1:** Baseline characteristics

	Group 1 *n* = 65	Group 2 *n* = 58	Group 3 *n* = 58	*p*-value*
Age, median (IQR)	66 (58–77)	73 (62–80)	70 (62–77)	a
BMI, median (IQR)	25 (24–26)	26 (23–28)	25 (23–28)	
Sex, *n* (%), male	36 (55.4)	37 (63.8)	42 (72.4)	b
PET-CT, *n* (%)	1 (1.5)	12 (20.7)	37 (63.8)	a, b, c
Charlson Comorbidity Index, ***n*** (%) 0–1 ≥ 2	39 (60.0) 20 (30.8)	35 (60.3) 23 (39.7)	35 (60.3) 23 (39.7)	
Tumor location, *n* (%) Upper 1/3 Middle Lower 1/3 Esophagogastric junction Linitis plastica	9 (13.8) 26 (40.0) 24 (36.9) 4 (6.2) 2 (3.1)	13 (22.4) 26 (44.8) 16 (27.6) 1 (1.7) 2 (3.4)	29 (50.0) 15 (25.9) 13 (22.4) 1 (1.7) 0	b, c
Gradeus, *n* (%) I II III Undefined	10 (15.4) 12 (18.5) 9 (13.8) 34 (52.3)	3 (5.2) 17 (29.3) 13 (22.4) 25 (43.1)	7 (12.1) 16 (27.6) 17 (29.3) 18 (31.0)	b
AJCC 8th edition TNM stage, *n* (%) 0 I II III IV	0 16 (24.6) 21 (32.3) 22 (33.8) 6 (9.2)	1 (1.7) 13 (22.4) 19 (32.8) 22 (37.9) 3 (5.2)	3 (5.2) 13 (22.4) 20 (34.5) 18 (31.0) 4 (6.9)	
Oncological treatment, *n* (%) No Preoperative or perioperative Preoperative chemoradiation Preoperative chemotherapy Perioperative chemotherapy Only preoperative chemotherapy Only postoperative	32 (49.2) 5 (7.7) 4 0 1 1 28 (43.1)	25 (43.1) 12 (20.7) 6 0 6 3 21 (36.2)	10 (17.2) 37 (63.8) 5 8 24 18 11 (19.0)	b, c
Surgical approach, *n* (%) Open Minimally invasive	64 (98.5) 1 (1.5)	48 (82.8) 10 (17.2)	9 (15.5) 49 (84.5)	a, b, c

a. p<0.05 between groups 1 and 2; b. p<0.05 between groups 1 and 3; c. p<0.05 between groups 2 and 3

### Outcomes

#### Primary Outcomes

**Fig. 2:**
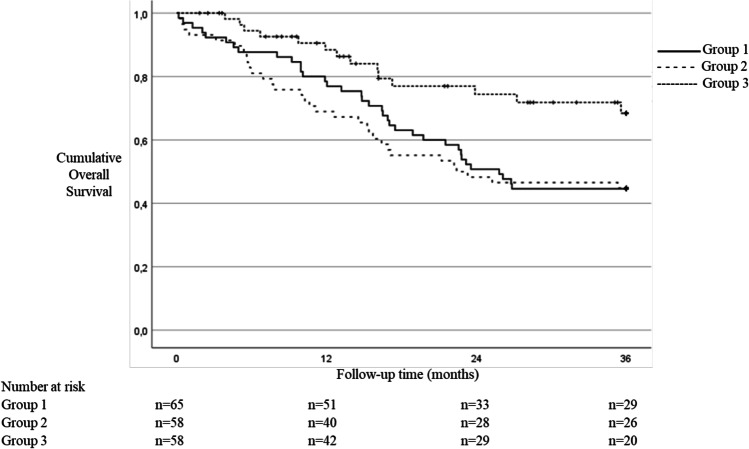
Overall 3-year survival in the study groups

Median lymph node yield increased from 17 in group 1 and 20 in group 2 to 23 in group 3 (*p* < 0.001). In these groups, the major complication rates were 12.3%, 32.8%, and 15.5% (group 1 vs. group 2, *p* = 0.007; group 1 vs. group 3, *p* = 0.736; group 2 vs. group 3, *p* = 0.018), respectively. Overall 1-year survival rates were 78.5%, 69.0%, and 90.2% (*p* = 0.018) and 3-year survival rates 44.6%, 44.8%, and 68.1% (*p* = 0.016), respectively (Fig. 2).

The crude and adjusted hazard ratios with 95% confidence intervals for overall 3-year mortality are presented in Table 2. Adjusted risk for overall 3-year mortality was HR 1.02, 95% CI 0.63–1.66 in group 2 and HR 0.37, 95% CI 0.20–0.68 in group 3 compared to group 1.

**Table 2:** Hazard ratios (HRs) with 95% confidence intervals (CIs) of overall 3-year mortality

	Number of patients	Group 1 *n* = 65 HR (95% CI)	Group 2 *n* = 58 HR (95% CI)	Group 3 *n* = 58 HR (95% CI)
Overall mortality (3 years)				
All patients (crude)	181	1.00 (Reference)	1.08 (0.67–1.74)	0.46 (0.25–0.85)
All patients (adjusted)*	181	1.00 (Reference)	1.02 (0.63–1.66)	0.37 (0.20–0.68)

^*^Adjusted for sex (male/female), age (< 75 years, ≥ 75 years), Charlson Comorbidity Index (0–1, ≥ 2), and pathological stage (0–I, II, and III–IV)

#### Secondary Outcomes

Surgical outcomes are presented in Table 3. The median blood loss decreased from 325 ml in group 1 and 400 ml in group 2 to 100 ml in group 3 (group 1 vs. group 3 and group 2 vs. group 3, *p* < 0.001). The rate of overall complications increased from 26.2% in group 1 to 44.8% in group 2 and decreased to 27.6% in group 3. The same trend was also seen in anastomotic leakage rates being 1.5%, 12.1%, and 3.4% in groups 1–3, respectively. In study group 2 and 3, there was no difference in the overall (34.7% vs. 37.3%) or major (20.4% vs. 28.4%) complication rates between neoadjuvant treated or non-treated patients (including also patients with postoperative treatment).

**Table 3:** Operative outcomes

	Group 1 *n* = 65	Group 2 *n* = 58	Group 3 *n* = 58	*p*-value*
Operative blood loss, ml, median (IQR)	325 (200–700)	400 (150–900)	100 (50–200)	b, c
Blood product utilization, *n* (%)	26 (40.0)	10 (17.2)	2 (3.4)	b, c
Hospital stay, days, median (IQR)	9 (7–12)	11 (8–14)	8 (7–10)	
Lymph node yield, *n*, median (IQR)	17 (11–23)	20 (12–24)	23 (17–35)	b, c
Resection margin, *n* (%) R0 R1 R2	56 (86.2) 3 (4.6) 6 (9.2)	51 (87.9) 2 (3.4) 5 (8.6)	51 (87.9) 6 (10.3) 1 (1.7)	
Dissection, *n* (%) D0 D1 D2 2-field	5 (7.7) 9 (13.8) 48 (73.8) 3 (4.6)	3 (5.2) 9 (15.5) 40 (69.0) 6 (10.3)	0 3 (5.2) 42 (72.4) 13 (22.4)	b, c
Complications, *n* (%) All Anastomotic leakage Minor (CDC I-II) Major (CDC IIIa-V)	17 (26.2) 1 (1.5) 7 (10.8) 8 (12.3)	26 (44.8) 7 (12.1) 6 (10.3) 20 (34.5)	16 (27.6) 2 (3.4) 7 (12.1) 9 (15.5)	a a a, c
Reoperation, 30 days, *n* (%)	6 (9.2)	13 (22.4)	6 (10.3)	
Mortality, *n* (%) 30 days 90 days	2 (3.1) 5 (7.7)	4 (6.9) 4 (6.9)	0 0	c b, c

a. *p* < 0.05 between groups 1 and; b. *p* < 0.05 between groups 1 and 3; c. *p* < 0.05 between groups 2 and 3

No change was detected in R0 resection or 30-day reoperation rates. The 30-day mortality rates between groups 1–3 were 3.1%, 6.9%, and 0% (group 1 vs. group 2, *p* = 0.326; group 1 vs. group 3, *p* = 0.178; group 2 vs. group 3, *p* = 0.042), respectively. The 90-day mortality rates were 7.7%, 7.4%, and 0% (group 1 vs. group 2, *p* = 0.866; group 1 vs. group 3, *p* = 0.031; group 2 vs. group 3, *p* = 0.042, respectively. Median hospital stay was 9 days (IQR 7–12) in group 1, 11 days (IQR 8–14) in group 2, and 8 days (IQR 7–10) in group 3.

#### Subgroup Analysis: Minimally Invasive vs. Open Surgery

In the comparison of short-term outcomes between minimally invasive and open surgery, the rate of major complication after minimally invasive surgery was 15.0% compared to 24.1% after open approach (*p* = 0.158). Difference in 90-day mortality was significant between minimally invasive and open approach (0% vs. 8.0%, *p* = 0.033). No difference in age or in the comorbidity burden between approaches was revealed. The median number of removed lymph nodes was significantly higher after minimally invasive approach (23 vs. 18, *p* = 0.043).

To estimate the potential role of learning curve effect on the increased rate of major complications in group 2, this group underwent further evaluation. Overall, patients in group 2 were significantly older than those in group 1 (Table 1). The increase in the rate of major complications from group 1 to group 2 was due to the increased complications after open surgery from 13.6 to 39.6% (*p* = 0.002). All anastomotic leaks in group 2 were after open surgery (0 vs. 14.6%). Within this group 2, difference in comorbidity burden with ≥ CCI 2 was significant between minimally invasive and open approach (10.0% vs. 45.8%, *p* = 0.035). The rate of neoadjuvant therapy was 40.0% before minimally invasive surgery compared to that of 16.7% (*p* = 0.098) before open approach.

## Discussion

This study indicates that the implementation of modern multimodal therapy combined with minimally invasive surgery in operable gastric cancer increases lymph node yield and improves overall 3-year survival without increasing postoperative morbidity in a medium-volume center.

The strength of this study is a consecutive series of histologically confirmed operable gastric and esophagogastric junction cancer in a medium-volume center without an apparent selection bias. Every patient that had GC related distal, subtotal, or total gastrectomy or esophagogastric resection was included into the study. After the introduction of minimally invasive approaches, only few patients were operated with an open approach. In learning phase of minimally invasive surgery, open approach was often used in older patients with more comorbidities having effect on postoperative outcomes after open surgery. Prospective data collection and double checking of the hospital records was performed. All patients were followed up in Central Finland Central Hospital for up to 5 years after surgery and nationwide compulsory databases enabled us to confirm a complete long-term mortality data. No patient was lost to follow-up. A major limitation is the small sample size, and, therefore, possible associations may be missed due to a lack of statistical power. Confidence intervals for reported hazard ratios are wide and replication studies are needed to confirm the findings. Another limitation of our study is the relatively short follow-up data. Therefore, only 3-year survival could be used as a long-term outcome.

In our population-based study including the elderly and patients with significant comorbidities, the rate of perioperative treatment was increased up to 63.8%. In a recent benchmark study, this rate was 51.3%.^27^ Real-life reports show that less than 27% GC patients have received guideline-based neoadjuvant therapy, and of them, 35% have not been able to complete it ^11^. The increased use of perioperative treatment can be considered a significant upgrade in GC care at our institution. Randomized studies have shown that preoperative chemotherapy improves 5-year survival in resectable stage II and III gastric cancer.^2,4–6,28^ One clear advantage of preoperative treatment is identification of patients with progressive disease avoiding futile surgery. Therefore, 3-year survival rising from 44.6 to 68.1% in this real-life series seems remarkably high but reasonable. An additional explanation to this improvement in long-term outcome is staging. The rate of PET-CT, although not sensitive in patients with mucinous or diffuse tumours, has increased simultaneously with the improved survival.^2,29^

In group 3, GC surgery was mostly performed by minimally invasive approach (84.5%). Compared with open surgery, it has oncological equivalent outcomes with numerous advantages, such as fewer complications, reduced blood loss, and shorter recovery time.^2,14,15^ Our short-term results with reduced blood loss mirror those of a recent meta-analysis including mostly Asian patients.^13^ In two recent European RCTs comparing laparoscopic and open gastrectomy after neoadjuvant therapy, no significant difference was detected in hospital stay, major or overall complication rates.^14,15^ Minimally invasive surgery is performed also with robot assisted technique. Although this could potentially provide advantages, scientific evidence is still lacking regarding its superiority.^30^ Ninety-day mortality in our study was, however, reduced after minimally invasive surgery for GC. In the group 3, short-term outcomes with overall complication rate of 27.6% and leakage rate of 3.4% are comparable with European benchmark values for GC surgery.^27,31^ In prospective study of 14,075 gastrectomy patients, leakage occurred more frequently after minimally invasive approach and during surgeon’s learning curve phase.^32^ The learning curve effect seems to be apparent in this series as well. During the implementation phase of minimally invasive surgery and multimodality therapy, major complication rates increased but those were, however, not associated with either minimally invasive surgery or multimodality therapy. During this period, older patients with an increased comorbidity burden were operated by open surgery. Therefore, the learning curve effect was associated to the whole process and not to the multimodal therapy or minimally invasive approach. Our previously reported excellent outcomes without detectable learning curve effect in minimally invasive esophageal cancer surgery could explain a technically easy transition from open to laparoscopic gastrectomy.^23^ With an increased experience, the rate of complication could potentially be reduced below those of open surgery.

In our study, lymph node yield reached the recommended minimum level in all the study groups being the highest with a median of 23 in group 3. The median of 23 was reached by minimally invasive surgery, as well. This median is significantly higher than that in Group 1 or that of 19 in our series of open D2 gastrectomies.^19^ The yield in a recent meta-analysis and two recent European RCTs was, however, comparable between open and laparoscopic approaches.^13–15^ In these studies, though not defined, 2D technology was most likely used. Modern 3D technology improves visibility and could potentially have a positive effect on surgical details. Overall, any concerns of the radicality of minimally invasive approach in advanced GC seem, even in a real-life medium-volume practise, unjustified.

## Conclusions

Based on the current study, a combination of guideline-based perioperative oncological treatment and minimally invasive surgery aiming to radical lymphadenectomy improves short- and long-term outcomes of operable gastric cancer.

## Data Availability

Anonymized data is available upon reasonable request from the corresponding author. Data sharing would need additional ethics committee approval.
